# Gastrointestinal Klippel–Trénaunay Syndrome Mimicking Ulcerative Colitis: A Case Report

**DOI:** 10.1155/carm/6780306

**Published:** 2026-07-21

**Authors:** Modar AlAli, Tarek Khazem, Hatem Yousef Almohamad, Hassan Habeeb Hasan, Ahmad Wassouf

**Affiliations:** ^1^ Faculty of Medicine, Damascus University, Damascus, Syria, damascusuniversity.edu.sy; ^2^ Gastroenterology Department, Faculty of Medicine, Al-Mouwasat University Hospital, Damascus University, Damascus, Syria, damascusuniversity.edu.sy

**Keywords:** anemia, colitis, diarrhea, gastrointestinal hemorrhage, iron deficiency, Klippel–Trénaunay–Weber syndrome, ulcerative, varicose veins

## Abstract

**Introduction:**

Klippel–Trénaunay syndrome (KTS) is a rare congenital syndrome characterized by the triad of capillary malformation, varicosities, and limb hypertrophy, with an incidence of 2‐5 per 100,000. Gastrointestinal (GI) involvement in KTS may be present in over 30% of patients, typically presenting with pain and bleeding. While bleeding is a well‐described symptom of GI involvement in KTS in the medical literature, diarrhea remains an uncommon and underreported manifestation. This report highlights this unique finding and the diagnostic complexity it presents.

**Case Presentation:**

A 64‐year‐old male presented to the hospital with recurrent foul‐smelling bloody diarrhea over the past two months. He had been previously diagnosed and treated for ulcerative colitis. Physical examination revealed pallor, macrodactyly, and segmental hypertrophy of the lower left limb. Investigations demonstrated severe iron‐deficiency anemia, splenomegaly, fundal varices, and a continuous 15‐cm colonic involvement from the anal verge with varicosities and bleeding. This led to the establishment of a diagnosis of KTS.

**Conclusion:**

This case highlights the diagnostic challenge that may arise from the complexity and variability of KTS presentations, which can mimic inflammatory bowel disease (IBD) due to findings like bloody diarrhea and extensive colonic involvement. Although diarrhea is rarely reported as a manifestation of KTS, it might result from existing GI vascular and lymphatic malformation, potentially leading to protein‐losing enteropathy. Awareness of this atypical presentation and early multidisciplinary evaluation are crucial for symptom control, preventing complications, and improving quality of life.

## 1. Introduction

Klippel–Trénaunay syndrome (KTS) is a rare and complex congenital syndrome characterized by the triad of capillary malformation (CM), varicosities, and hypertrophy of the affected limb [1], with an estimated incidence of 2–5 per 100,000 individuals [2]. While cutaneous vascular malformations and unilateral hypertrophy are the hallmark features, visceral manifestations, like gastrointestinal (GI) involvement and splenomegaly, carry the risk of serious complications [3, 4]. GI involvement may be present in over 30% of patients and mainly present as an edematous, thickened colorectum caused by an extensive venous malformation (VM) [5].

Many complications may arise from GI involvement including pain, bleeding, and less commonly diarrhea [2]. Lower GI bleeding is the predominant complication of GI involvement, especially the rectosigmoid colon in KTS, varying from occult hemorrhage to critical life‐threatening episodes of bleeding [6]. This recurrent bleeding can cause iron‐deficiency anemia and can be a substantial source of morbidity and mortality in KTS patients with GI involvement [3, 5, 7].

We describe the case of an older male with KTS presenting with recurrent bloody diarrhea initially misdiagnosed as ulcerative colitis (UC). This case underscores the diagnostic challenge posed by the overlap of KTS with more common GI diseases and highlights the importance of early recognition to guide management.

## 2. Case Presentation

A 64‐year‐old male presented to the hospital with a 2‐month history of recurrent foul‐smelling bloody diarrhea. The patient was initially diagnosed with UC based on chronic bloody diarrhea and endoscopic findings of continuous mucosal inflammation.

He had been previously diagnosed with UC five years ago at a local hospital and treated with azathioprine, mesalamine, pantoprazole, and methylprednisolone. His past history included jejunostomy and partial colectomy to manage a bleeding duodenal ulcer and a severe lower GI bleeding, respectively.

**FIGURE 1:**
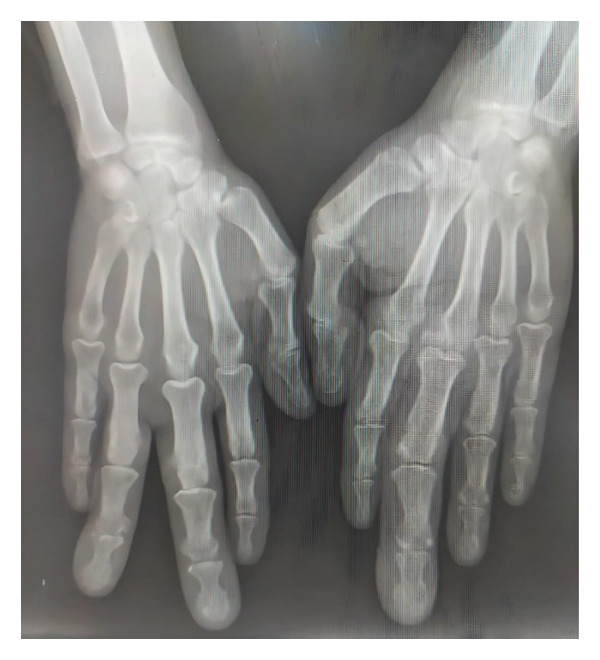
An X‐ray showing macrodactyly of both hands.

**FIGURE 2:**
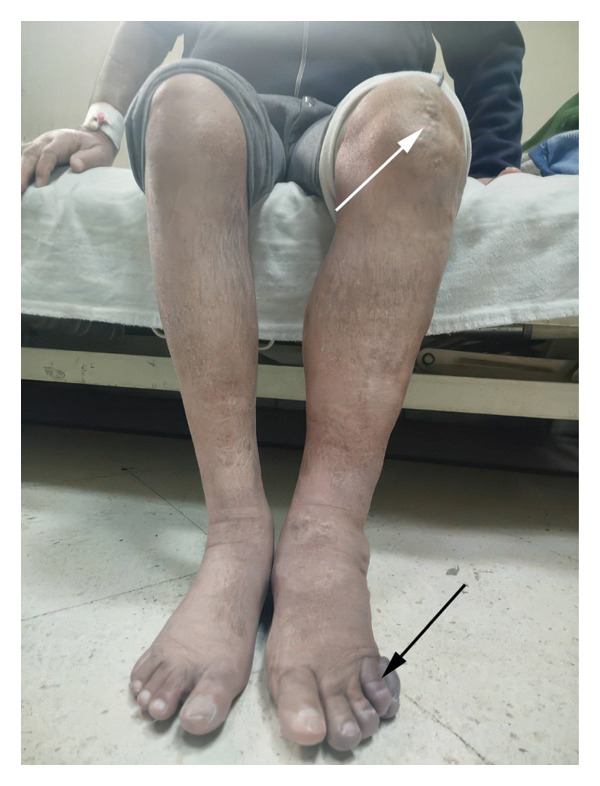
A photograph of the lower limbs showing segmental hypertrophy of the left lower limb, along with varicose veins (white arrow) and hemangiomas (black arrow).

On examination, he exhibited conjunctival pallor, macrodactyly of the hands (Figure 1), and hemangiomas, varicose veins, and segmental hypertrophy with nonpitting lymphedema of the left lower limb, as depicted in Figure 2. These anomalies had been present since birth. Digital rectal exam showed blood staining. Additional laboratory results demonstrated a severe iron‐deficiency anemia (hemoglobin of 6.5 g/dL, low ferritin, and low transferrin saturation).

**FIGURE 3:**
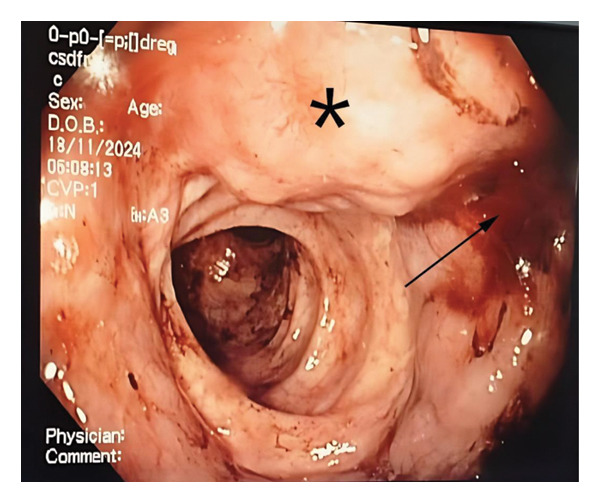
An endoscopic photograph showing signs of bleeding (black arrow) associated with venous dilation (black asterisk).

Abdominal ultrasound revealed moderate splenomegaly with normal liver echotexture. Moreover, Doppler ultrasound confirmed left saphenous vein insufficiency with no signs of thrombosis. Upper GI endoscopy revealed isolated fundal varices, whereas subsequent colonoscopy showed active bleeding as well as extensive venous dilations extending 15 cm from the anal verge, demonstrated in Figure 3. Biopsy findings were not consistent with UC. Finally, an abdominal CT scan revealed dilated venous vasculature in the hepatic and splenic hila, left internal iliac, and left renal veins, as well as rectal mucosal thickening with calcifications. Taken together, these findings supported the diagnosis of KTS. The patient underwent endoscopic argon laser coagulation of the vascular lesions and was scheduled for follow‐up.

## 3. Discussion

KTS was first described in 1900 as a sporadic malformation syndrome characterized by a triad of cutaneous CM, varicosities, and asymmetric hypertrophy of bone and overlying soft tissues [2, 8]. Although several theories were proposed regarding its pathogenesis [9], recent evidence classified KTS within the PIK3CA‐related overgrowth spectrum (PROS), together with other overgrowth syndromes like CLOVES and fibroadipose hyperplasia. All are associated with somatic heterozygous gain‐of‐function mutations in the PIK3CA gene, presented in a mosaic pattern [8]. The heterogeneity of these clinical manifestations creates diagnostic challenges and leads to overlap with other disorders such as Proteus syndrome and Parkes Weber syndrome [9].

According to the diagnostic criteria proposed by Oduber et al., KTS is marked by 2 major groups of features: congenital vascular malformations and disturbed growth. The presence of either CMs (like port‐wine stains) or VMs (like varicosities, hypoplasia or aplasia of veins, and valvular malformations) is essential to establish the diagnosis. Disturbed growth can occur in many forms, including hypertrophy of a small body part (such as macrodactyly) or a large region such as the lower limb; these anomalies can be observed at both the location of the vascular malformation and other sites [9]. In our patient, varicosities, lymphatic malformations, macrodactyly, and segmental hypertrophy supported the clinical diagnosis.

KTS may affect visceral organs such as the spleen, liver, bladder, and colon leading to serious complications [2]. GI involvement usually affects the colorectum, presenting as a thickened, edematous mucosa due to the VM networks around and within the bowel wall [5]. These VMs are frequently observed in two adjacent regions but arise from distinct underlying mechanisms: anorectal and anorectosigmoid VMs. Anorectal VMs are linked to internal iliac vein (IIV) malformations causing pelvic venous hypertension. It commonly manifests as thickened edematous rectum with the possibility of bleeding [5], which might be misinterpreted as internal hemorrhoids [10], while anorectosigmoid VMs, which involve the upper rectum and the sigmoid colon, drain into both the inferior mesenteric vein (IMV) and the IIV. This dual reflux can lead to venous stasis, thrombosis, portal hypertension, and splenomegaly [5]. Our case demonstrated features consistent with the anorectosigmoid type, including fundal varices and splenomegaly. Additionally, the venous ectasia involving the splenic and hepatic hila, as well as the left IIV, is consistent with the dual reflux to IMV and IIV [3].

These vascular abnormalities commonly result in recurrent rectal bleeding, reported in 2%–23% of KTS patients, and may range in severity from asymptomatic occult bleeding to massive hemorrhage [5, 11]. Chronic blood loss can cause severe iron‐deficiency anemia, with hemoglobin levels reported as low as 2.5 and 5.7 g/dL. Symptoms include fatigue, shortness of breath, and dizziness, with examination revealing pallor of the conjunctiva and skin and tachycardia [3, 7]. While bleeding is well recognized, diarrhea is rather a unique manifestation of KTS. Only a few reports have mentioned it [1, 2, 12]. One plausible mechanism is that protein‐losing enteropathy secondary to intestinal lymphangiectasia, compounded by venous dilation, can cause diarrhea [2]. In our patient, diarrhea was the main complaint, and the existence of abnormal findings in the colonic mesentery was supportive of this hypothesis.

In conjunction with clinical findings, imaging techniques are used to establish the diagnosis. Doppler ultrasound, MRI, and CT scan can be very informative in assessing vascular and lymphatic anomalies [7], while plain radiographs may reveal bone abnormalities [13]. For GI involvement in KTS, colonoscopy is considered the gold standard to identify precise localization and extent of mucosal vascular lesions [6, 10]. Endoscopic findings range from multiple venous varicosities [10] to continuous circumferential submucosal involvement of the rectum and sigmoid [14]. It is also important to emphasize that biopsies are not recommended due to the risk of uncontrollable bleeding [3].

UC typically presents with bloody diarrhea, anemia, fatigue, and continuous circumferential involvement on colonoscopy, with biopsies being the common practice for definitive diagnosis [15]. Some GI manifestations of KTS may closely mimic UC, with overlapping symptoms and endoscopic findings, potentially leading to misdiagnosis and inappropriate therapy [11, 16]. Several reports have described this diagnostic pitfall, which, combined with the higher prevalence of UC, likely initially overshadowed the clinical association with cutaneous lesions and limb hypertrophy in our case [6, 10]. This overlap emphasizes the importance of recognizing the multisystem nature of KTS, particularly when GI symptoms, such as “colitis,” present alongside vascular malformation and limb anomalies. The presence of a recurrent bloody diarrhea as well as common endoscopic findings like the continuous circumferential involvement of the distal colon, along with bleeding, further complicated the diagnosis of KTS mimicking UC.

Management of KTS is complex and best approached by a multidisciplinary team. While there is no definitive treatment for KTS, a conservative symptomatic approach can help improve patients’ quality of life. Special complications may require specific management, like localized intravascular coagulopathy, surgical resection of the involved bowel bleeding [17], and laser therapy [2]. Additionally, sirolimus has demonstrated potential in improving symptoms and managing vascular malformations [18].

Finally, KTS can manifest with very diverse findings, and its visceral involvement may mimic different pathologies. Precise and early diagnosis is crucial and can protect the patient from complex complications and ineffective treatments. This can be achieved through careful clinical examination and targeted radiological investigations in order to reduce the risk of misdiagnosis.

## Author Contributions

Hatem Yousef Almohamad and Hassan Habeeb Hasan contributed to conceptualization and design of the case report. They were responsible for the patient’s clinical management and data curation, including collection of clinical and imaging data. Modar AlAli and Hatem Yousef Almohamad performed the formal analysis and interpretation of the findings. Modar AlAli and Tarek Khazem contributed to writing–original draft preparation. Hatem Yousef Almohamad and Hassan Habeeb Hasan contributed to writing–review and editing, providing critical revision for important intellectual content. Ahmad Wassouf provided supervision and overall guidance throughout the project. Modar AlAli was responsible for project administration, manuscript submission, and correspondence with the journal.

## Funding

This research received no specific grant from any funding agency in the public, commercial, or not‐for‐profit sectors.

## Disclosure

All authors have read and approved the final version of the manuscript.

## Ethics Statement

The authors have nothing to report.

## Consent

Written informed consent was obtained from the patient and their legal guardian for the publication of this case report and any accompanying images. This study complies with the ethical guidelines outlined by the Committee on Publication Ethics (COPE).

## Conflicts of Interest

The authors declare no conflicts of interest.

## Supporting Information

Additional supporting information can be found online in the Supporting Information section.

## Supporting information


**Supporting Information** The CARE reporting checklist.

## Data Availability

The data that support the findings of this study are available on request from the corresponding author. The data are not publicly available due to privacy or ethical restrictions.

## References

[1] Vazquez-Sequeiros E. , Sorbi D. , Kamath P. S. , and Wiersema M. J. , Klippel-Trenaunay-Weber Syndrome: Role of EUS, Gastrointestinal Endoscopy. (2001) 54, no. 5, 660–661, 10.1067/mge.2001.116323. 11677496

[2] Asghar F. , Aqeel R. , Farooque U. , Haq A. , and Taimur M. , Presentation and Management of Klippel-Trenaunay syndrome: a Review of Available Data, Cureus. (2020) 10.7759/cureus.8023. PMC7282379 32528762

[3] Tetangco E. P. , Arshad H. M. S. , and Silva R. , Klippel-Trenaunay syndrome of the Rectosigmoid Colon Presenting as Severe Anemia, ACG Case Reports Journal. (2016) 3, no. 1, 10.14309/crj.2016.134. PMC5126491 27921060

[4] Misawa T. , Shiba H. , Fujiwara Y. et al., Massive Splenomegaly Caused by Cavernous Hemangiomas Associated with Klippel–Trenaunay syndrome: Report of a Case, Surgery Today. (2014) 44, no. 1, 197–200, 10.1007/s00595-013-0779-y. 24254059

[5] Wang H. , Lin W. , Xie C. , Yang W. , Zhou J. , and Guo Z. , Gastrointestinal Involvement in Klippel-Trénaunay syndrome: Pathophysiology, Evaluation, and Management, Orphanet Journal of Rare Diseases. (2023) 18, no. 1, 10.1186/s13023-023-02857-5. PMC10496303 37700367

[6] Li T. , Hu S. Y. , Chen Z. T. , Chen Z. Q. , and Zhi X. T. , Colorectal Cavernous Hemangioma in Klippel-Trenaunay syndrome: a Rare Cause of Abdominal Pain and Hematochezia, Surgery. (2015) 157, no. 2, 402–404, 10.1016/j.surg.2013.08.006. 25759876

[7] Al-Anbagi U. , Elmakaty I. , Al-Zoubi H. M. , Nashwan A. J. , and Sharif M. , Klippel-Trenaunay syndrome: a Case Study of Severe Anemia in a Rare Vascular Disorder, Cureus. (2024) 10.7759/cureus.75725. PMC11732612 39816300

[8] Vahidnezhad H. , Youssefian L. , and Uitto J. , Klippel–Trenaunay syndrome Belongs to the *PIK3CA* ‐Related Overgrowth Spectrum (PROS), Experimental Dermatology. (2016) 25, no. 1, 17–19, 10.1111/exd.12826. 26268729

[9] Oduber C. E. U. , Van Der Horst C. M. A. M. , and Hennekam R. C. M. , Klippel-Trenaunay syndrome: Diagnostic Criteria and Hypothesis on Etiology, Annals of Plastic Surgery. (2008) 60, no. 2, 217–223, 10.1097/SAP.0b013e318062abc1. 18216519

[10] Khan Z. , Muqim R. U. , Zarin M. , and Asad M. , Klippel-Trénaunay Syndrome With Bleeding per Rectum as a Major Surgical Concern: A Case Report, Journal of Medical Science. (2021) 28, no. 4, 384–386, 10.52764/jms.20.28.4.18.

[11] Wang Z. , Wang X. , Zhao Q. et al., Klippel–Trenaunay syndrome with Multiorgan Vascular Involvement and Gastrointestinal Bleeding: a Case Report and Literature Review, Medicine (Baltimore). (2025) 104, no. 8, 10.1097/MD.0000000000041634. PMC11856890 39993099

[12] Jacob A. G. , Driscoll D. J. , Shaughnessy W. J. , Stanson A. W. , Clay R. P. , and Gloviczki P. , Klippel-Trénaunay syndrome: Spectrum and Management, Mayo Clinic Proceedings. (1998) 73, no. 1, 28–36, 10.1016/S0025-6196(11)63615-X. 9443675

[13] Obara P. , McCool J. , Kalva S. P. et al., ACR Appropriateness Criteria® Clinically Suspected Vascular Malformation of the Extremities, Journal of the American College of Radiology. (2019) 16, no. 11, S340–S347, 10.1016/j.jacr.2019.05.013. 31685102

[14] Sepulveda A. , Soriano H. , and Espino A. , Gastrointestinal Tract Involvement in Klippel–Trénaunay syndrome, The Lancet Gastroenterology & Hepatology. (2018) 3, no. 7, 10.1016/S2468-1253(18)30140-7. 29893236

[15] Ungaro R. , Mehandru S. , Allen P. B. , Peyrin-Biroulet L. , and Colombel J. F. , Ulcerative Colitis, The Lancet. (2017) 389, no. 10080, 1756–1770, 10.1016/S0140-6736(16)32126-2. PMC6487890 27914657

[16] Deepinder F. , GI Bleeding, Colon Varicosities, and Visceral Enlargement as a Manifestation of Klippel–Trenaunay syndrome, Clinical Gastroenterology and Hepatology. (2011) 9, no. 12, e126–e127, 10.1016/j.cgh.2011.06.023. 21723229

[17] John P. R. , Klippel-Trenaunay syndrome, Techniques in Vascular and Interventional Radiology. (2019) 22, no. 4, 10.1016/j.tvir.2019.100634. 31864529

[18] Hammill A. M. , Wentzel M. , Gupta A. et al., Sirolimus for the Treatment of Complicated Vascular Anomalies in Children, Pediatric Blood and Cancer. (2011) 57, no. 6, 1018–1024, 10.1002/pbc.23124. 21445948

